# Developmental Course and Risk Factors of Physical Aggression in Late Adolescence

**DOI:** 10.1007/s10578-020-01049-7

**Published:** 2020-08-27

**Authors:** Marit Henriksen, Marit Skrove, Gry Børmark Hoftun, Erik R. Sund, Stian Lydersen, Wan-Ling Tseng, Denis G. Sukhodolsky

**Affiliations:** 1 Department of Clinical and Molecular Medicine, Faculty of Medicine and Health Science, The Norwegian University of Science and Technology, 7491 Trondheim, Norway; 2 Department of Public Health and Nursing, Faculty of Medicine and Health Science, The Norwegian University of Science and Technology, Trondheim, Norway; 3 Department of Mental Health, Faculty of Medicine and Health Science, The Norwegian University of Science and Technology, Trondheim, Norway; 4 Child and Adolescent Psychiatric Clinic, St. Olav’s University Hospital, Trondheim, Norway; 5 Children’s Clinic, St Olav’s University Hospital, Trondheim, Norway; 6 Faculty of Nursing and Health Sciences, Nord University, Levanger, Norway; 7 Nord-Trøndelag Hospital Trust, Levanger Hospital, Levanger, Norway; 8 Child Study Center, Yale University School of Medicine, New Haven, CT USA

**Keywords:** Physical aggression, Physical fighting, Adolescence, Risk factors, Longitudinal

## Abstract

This study examined risk factors of physical aggression during transition from early to late adolescence using a two-wave longitudinal study. Specifically, we examined if risk factors in early adolescence predict physically aggressive behavior starting in late adolescence and why some adolescents desist physical aggressive behavior while others do not. The study sample consisted of 2289 Norwegian adolescents (1235 girls) who participated in the Young-HUNT1 study (mean age 14.5) and the follow-up study 4 years later, Young-HUNT2 study (mean age 18.4). One in six young adolescents reported engaging in physical fights. Moreover, physical aggression in early adolescence was significantly associated with male gender, attention problems, academic problems, being bullied, drinking alcohol, and smoking. Male gender and heavy drinking during early adolescence increased the risk for newly emerging aggressive behavior in late adolescence, whereas heavy drinking during early adolescence was a predictor for persistent versus desisting aggressive behavior in late adolescence.

## Introduction

Physical aggression in childhood and adolescence represents a major clinical and public health concern. Thus, in a population study in the United States in 2017, 23.6% of high school students (grade 9 to 12) reported participating in a physical fight in the preceding year [1]. At the global level, interpersonal violence is the fourth leading cause of death among adolescents aged 15 to 19, with a 5.5% mortality rate [2]. Physical aggression and delinquency can have long lasting negative consequences for the perpetrators and the victims. Perpetrators of physical aggression are shown to have an increased risk for later physical violence and crimes [3, 4], school drop-out [4, 5], substance abuse [5], future mental, and physical health problems and economic difficulties [6]. Victims, in addition to the physical injuries, have an increased risk of mental health problems, educational and economic difficulties, involvement in crimes [7], and externalizing problems [8].

From a developmental perspective, the type and prevalence of physically aggressive behavior change with age. In typically developing youth, the prevalence of physical aggression peak at around age 3 and decrease from childhood to adolescence, as children acquire more advanced cognitive, social and language skills, and learn to regulate emotions and use alternative conflict resolution strategies [9–11]. Some children continue to display high levels of physical aggression throughout their childhood and adolescence [12–14]. Nagin and Tremblay [3], in a sample of 1037 boys followed from age 6 to 15 years old, identified four trajectories of physical aggression: *chronic/persisting aggression* (4%), *high near-desisting* (28%), *moderate desisting* (52%) and *no physical aggression trajectory* (17%). Since this seminal study, these developmental trajectories of physical aggression have been well replicated in other studies [10, 13, 15, 16], although the number of trajectories and number of children in each category varies as a function of sample characteristics and aggression measurements.

Empirical studies have identified multiple correlates and risk factors of physical aggression in adolescence [17, 18]. Both girls and boys can initiate aggressive behavior, but boys display more physical aggression than girls [9, 11, 13, 16]. Mental health disorders are associated with physical aggression, most notably disruptive behavior disorders and attention-deficit/hyperactivity disorder (ADHD), but also anxiety and depression [19–21]. Longitudinal studies have found ADHD symptoms to predict physical aggression [3, 5, 22], physical aggression to predict depression [23, 24], and anxiety to protect against aggression [25]. Physical aggression is also associated with academic difficulties [26, 27], and low self-esteem [28]; however, some research indicates that high self-esteem may also be associated with physically aggressive behavior [29].

Regarding social factors, family characteristics including single-parent household and divorced parents have been associated with elevated risk for physical aggression [10, 16, 17, 30]. While some studies present evidence that having divorced parents is a risk factor for later physical aggression [31, 32], others do not [10]. Peer factors, such as peer rejection, bullying, and loneliness, commonly co-occur with physical aggression in adolescence [33–36], and bullying victimization in adolescence has been linked to later violence [37].

During adolescence, developmentally normative changes in social relationships, including decreasing parental supervision and increasing influence of peers, may also elevate risk for aggression [31, 38]. Similarly, engagement in new, risky behaviors may impact aggressive behavior. For example, increases in alcohol drinking and intoxication, drug use and smoking have been associated with physical aggression in adolescence [5, 39–42]. In a study on alcohol consumption and aggression (verbal and physical) within a large sample of college students (n = 6282), a direct relationship between alcohol and aggression was found, indicating that increased alcohol use increases risk for aggression [43].

Compared to the large number of studies examining risk factors of physical aggression in childhood and adolescence, much less is known about the risk factors of physical aggression in late adolescence, during the important transition to adulthood. Longitudinal studies of aggression that follow children into young adulthood often focus on broad categories of delinquency [44, 45], rather than specific acts of physical aggression such as fighting. However, studies on physical aggression in childhood and adolescence have shown that physical aggression is a specific and separate dimension of disruptive behavioral problems [46], and physical aggression in late adolescence and young adulthood can also occur outside of delinquency trajectories, for example in relation to intimate partner violence [47]. In addition, few studies have examined the risk factors and courses of physical aggression from early to late adolescence. During this time, adolescents are faced with important developmental tasks, such as identity formation, separating from parents, and developing autonomy and intimacy with others [48, 49] at the same time as parents’ supervision decreases. Although physical aggression generally declines with increasing age, the specific characteristics of the adolescent period may place some individuals at risk for the onset of physically aggressive behavior during this developmental phase. Two studies support the trend of increasing physically aggressive behavior over the course of adolescence [15, 31]. However, one of these studies did not examine risk factors of courses of aggression, and the other study used a broader measure of aggression that did not distinguish between physical and verbal aggression (e.g., verbal threats).

### The Aims of the Study

This study addresses these gaps in the literature by examining risk factors of physical aggression over a key developmental stage, early to late adolescence. Using a population-based prospective, longitudinal cohort study, we measured a common form of physical aggression in adolescents—fighting among peers—at two timepoints four years apart, corresponding to early (13–16) and late adolescence (17–20). The consequences of participating in physical fighting differs by age. The objective of the study was to examine the correlates of this serious and dangerous form of aggression in early adolescence and its course from early to late adolescence. We wanted to examine if known risk factors, anxiety and depression, loneliness, self-esteem problems, attention problems, reading and writing problems, being bullied, parent’s marital status, alcohol intoxication, and smoking, in early adolescence can predict why some adolescents have an onset of physical aggression in late adolescence, as this group has received less focus in the literature. We also wanted to examine if the same risk factors can explain why aggressive behavior desists in some adolescents and not others. From previous studies we know that a small group of children display physical aggressive behavior throughout childhood, adolescence, and into adulthood [12–14]. Identifying risk factors that separate persisting from desisting physical aggression in adolescence, is important for the development of efficient interventions hindering physical aggressive behavior into adulthood. Higher cumulative risks are associated with higher levels of physical aggression [50, 51]. The prospective, longitudinal cohort study, The Young-HUNT study, offers a unique opportunity to use a large non-clinical adolescent population of boys and girls to investigate several risk factors and how they affect courses of physical aggression in adolescence.

## Methods

### Study Population and Design

All residents aged 13 years and older in the county of Nord-Trøndelag, Norway were invited to participate in The Nord-Trøndelag Health Study (HUNT) that was conducted between 1995 and 1997 [52, 53]. The population was stable and homogenous and therefore suitable for epidemiological, longitudinal studies [54]. Adolescents aged 13 to 19 years old were invited to participate in Young-HUNT1 (YH1), the first wave (time 1) of the study (response rate 88.1%). Adolescents who were 13–16 years old in YH1 were then invited to participate in the follow-up study Young-HUNT2 (YH2) four years later (time 2). A total of 2399 adolescents participated (response rate 76.8%) in both waves. The sample for the present study consisted of 2289 subjects. Subjects with missing values on the dependent variable (physical fighting) (n = 110) were excluded. The study is Data Protection Impact Assessment (DPIA) approved by The Norwegian University of Science and Technology (NTNU) and approved by the Regional Committee for Medical and Health Research Ethics (REK) in Norway (ref. 2017/1208/REK-midt).

### Measures

At time 1 and time 2, the subjects completed a self-report questionnaire in a classroom setting at school. Subjects not in school got the questionnaire by mail and were asked to fill out and return it. The questionnaire included questions about physical and psychological health, family variables, lifestyle, use of medication and health service, school difficulties, and pubertal status. The original questionnaire translated to English is available at the HUNT Web page: (https://www.ntnu.edu/hunt/data/que).

#### Dependent Variable

To study physical aggression, adolescents were asked to indicate whether they had been in a physical fight using a four-point response scale (*Never, Sometimes, Often,* and* Very often*). The item was a part of the 14-item School Adjustment Questionnaire (designed by the Norwegian Institute of Public Health) assessing behaviors at school or in relation to school. Although the time frame was not specified in the item itself, this item was embedded in the questionnaire about current health-related issues that were queried for the period of the past 12 months. It was therefore assumed that children rated their aggressive behavior for this period. The scale`s psychometric properties and validation are described in previous studies [55, 56]. The majority of adolescents in YH1 and YH2 endorsed *Never* on this question (see Table 1). Of the 2289 participants, 23 children (1.0%) endorsed *Often* and 9 (0.4%) children endorsed *Very often* in YH1; in YH2, 12 children (0.5%) endorsed *Often* and 11 (0.5%) children endorsed *Very often.* Due to the highly skewed distribution, the physical aggression variable was dichotomized as *No aggression* for all subjects who responded *Never* and *Physical aggression* for all subjects who endorsed *Sometimes, Often*, or *Very often*. To examine the developmental course of aggression, we used the dichotomized physical aggression variable in YH1 and YH2 and generated a variable with four courses of physical aggression: (1) the non-aggression group (no physical fighting in YH1 or YH2), (2) the desisting aggression group (physical fighting in YH1, but not YH2), (3) the late adolescent aggression group (physical fighting in YH2, but not YH1), and (4) the persistent aggression group (physical fighting in YH1 and YH2).

**Table 1:** Prevalence of physical fighting inYH1 and YH2

	PF in YH2				
	Never	Sometimes	Often	Very often	Total
PF in YH1					
Never	1793	89	5	4	1891 (82.6%)
Sometimes	271	86	4	5	366 (16.0%)
Often	10	9	2	2	23 (1.0%)
Very often	6	2	1	0	9 (0.4%)
Total	2080 (90.9%)	186 (8.1%)	12 (0.5%)	11 (0.5%)	2289 (100%)

*YH1* Young-HUNT1, *YH2* Young-HUNT2, *PF* physical fighting

#### Independent Variables

##### Mental Health Variables

Symptoms of anxiety and depression were assessed using a shortened and previously validated five-item scale (SCL-5) [57, 58] derived from the Hopkins Symptom Checklist for anxiety and depression (SCL-25). The subjects were asked to rate, on a four-point scale (*Never, Sometimes, Often,* and *Very often*) how much they had been bothered by the following thoughts and feelings during the last 14 days: *Been constantly afraid and anxious, Felt tense or uneasy, Felt hopelessness when you think of the future, Felt dejected or sad,* and *Worried too much about various things.* The items were summed and averaged, with higher scores representing more symptoms of anxiety and depression. Cronbach`s alpha for SCL-5 was 0.77. Loneliness was indexed by the item *Do you feel lonely?* on a five-point scale (*Very often, Often, Sometimes*, *Seldom,* and *Very seldom or never*).

A short version of the Rosenberg Self-Esteem Scale [59, 60] was used to measure self-esteem. The subjects were asked to rate on a four-point scale (*Strongly agree, Agree, Disagree,* and *Strongly Disagree*) the following four items: *I take a positive attitude toward myself, I certainly feel useless at times, I feel I do not have much to be proud of,* and *I feel that I`m a person of worth, at least on an equal plane with others*. The positively phrased items were reverse scored. The items were summed and averaged, with higher scores representing lower self-esteem. Cronbach`s alpha for the Self-esteem scale was 0.71.

##### School Variables

Items from the School Adjustment Questionnaire with a 4-point response scale (*Never, Sometimes, Often,* and* Very often*) were used to index attention problems and bullying. Attention problems were assessed by two items: *Have difficulties concentrating during class* and *Can`t manage to be calm/ sit still during class*. The two items were averaged, with higher scores representing more problems with attention. Experience with being bullied was assessed by one item: *Are teased/harassed by other students?* Subjects were asked *How often do you feel your reading or writing skills are below the level of the tasks you do at school and/or in your spare time?* (separate item for reading and for writing) with a five-point response scale (*Never, Very seldom, Sometimes, Often,* and *Always)*. The items were summed and averaged, with higher scores representing more problems with reading and writing.

##### Family Variable

Subjects were asked *Are your parents separated or divorced, or have they lived separately for more than one year?* Parent’s marital situation was dichotomized into *Parents living together* and *Parents separated or divorced.*

##### Substance Use

Subjects were asked *Have you ever drunk so much alcohol that you felt intoxicated (drunk)?* with a 5-point response scale (*No, never, Yes, once, Yes, 2–3 times, Yes, 4–10 times, and Yes, more than 10 times*). Responses were further categorized into three categories: *Never been drunk, Been drunk 1 to 10 times,* and *Been drunk more than 10 times.* Subjects were also asked *Do you smoke?* and were defined as current smokers if they responded *Yes* to smoking daily or occasionally.

### Analyses

The distribution of the four categories of responses to the physical fighting variable in YH1 and YH2 is shown in a cross tabulation. In the remaining analyses, the three categories *Sometimes, Often,* and* Very often* were merged, giving the dichotomy *aggressive* versus *non-aggressive*. Descriptive statistics for the correlates are displayed for the total sample in the study, the aggressive vs the non-aggressive adolescents in YH1, and the four courses of physical aggression. The correlates used here and in the later analyses were: mental health variables (anxiety and depression symptoms, loneliness, and self-esteem problems), school variables (attention problems, reading- and writing problems, and being bullied), parent’s marital status, and substance use (alcohol intoxication and smoking), all from YH1.

In a cross-sectional analysis, we used logistic regression with physical aggression in YH1 as dependent variable and correlates in YH1. The correlates were first examined one at a time only adjusted for the covariates age and gender (Model 1), and then jointly (Model 2). The course of physical aggression was studied using multinomial logistic regression with the four courses of physical aggression from YH1 to YH2 as a four-category dependent variable. The OR in multinomial logistic regression has the same interpretation as the OR in binary logistic regression. Risk factors were first examined independently, only adjusted for age and gender (Model 1), and then all included for a fully adjusted model (Model 2). Choice of reference group in the analyses were based on the aim being studied. When examining what separates *the non-aggression group* from *the late adolescent aggression group*, in terms of risk factors, the *non-aggression group* was the reference group. When examining what separates *the desisting aggression group* from *the persistent aggression group, the desisting aggression group* was the reference group. Two-sided p values < 0.05 were used to indicate statistical significance. Missing values were handled using available case analysis, that is, each analysis included the cases with available data on the variables included in the analysis. All statistical analyses were conducted using Stata for Windows, version 15 (STATA Corp, College Station, TX, USA).

## Results

### Prevalence of Physical Aggression

The sample consisted of 1235 girls (46.1%) and 1054 boys (54.0%). Mean age was 14.5 years (SD 0.91) in YH1 (Time 1), and 18.4 years (SD 0.80) in YH2 (Time 2). In YH1, 1891 (82.6%) subjects responded that they *Never* participate in physical fights while 398 (17.4%) responded *Sometimes*, *Often* or *Very often*. In YH2, 2080 (90.9%) subjects responded that they *Never* participate in physical fights while 209 (9.1%) responded *Sometimes*, *Often* or *Very often*. (see Table 1). Higher percentage of boys engaged in physical aggression than girls at both timepoints (YH1—85.9% versus 14.1%, YH2—82.3% versus 17.7%).

Of the 2289 adolescents, 1793 adolescents (78.3%) were in *the non-aggressive group*, 287 adolescents (12.5%) were in *the desisting aggression group*, 98 adolescents (4.3%) were in *the late adolescent aggression group*, and 111 adolescents (4.9%) were in *the persistent aggression group*. The sex distribution was 1151 girls (64.2%) versus 642 boys (35.8%) in *the non-aggression group*, 47 girls (16.4%) versus 240 boys (83.6%) in *the desisting aggression group*, 28 girls (28.6%) versus 70 boys (71.4%) in *the late adolescent aggression group*, and 102 boys (91.9%) versus 9 girls (8.1%) in *the persistent aggression group*. See Table 2 for more characteristics of the study subjects.

**Table 2:** Sample characteristics and risk factors in YH1 by total sample, physical aggression and no-aggression in YH1, and four courses of physical aggression

	Total sample	PA in YH1	No PA in YH1	The non-aggression group	The desisting aggression group	The late aggression group	The persistent aggression group
	Mean (SD) or n (%)	Mean (SD) or n (%)	Mean (SD) or n (%)	Mean (SD) or n (%)	Mean (SD) or n (%)	Mean (SD) or n (%)	Mean (SD) or n (%)
Participants (n = 2289)	2289	398	1891	1793 (78.3)	287 (12.5)	98 (4.3)	111 (4.9)
Age^a^ (n = 2289)	14.51 (0.91)	14.39 (0.89)	14.54 (0.91)	14.54 (0.91)	14.38 (0.86)	14.44 (0.85)	14.42 (0.96)
Sex^b^ (n = 2289)							
Girls	1235 (46.0)	56 (14.1)	1179 (62.3)	1151 (64.2)	47 (16.4)	28 (28.6)	9 (8.1)
Boys	1054 (54.0)	342 (85.9)	712 (37.7)	642 (35.8)	240 (83.6)	70 (71.4)	102 (91.9)
Anxiety and depression^a^ (n = 2282)	1.37 (0.43)	1.38 (0.44)	1.37 (0.43)	1.37 (0.43)	1.39 (0.44)	1.36 (0.45)	1.37 (0.46)
Loneliness^a^ (n = 2281)	1.93 (0.89)	1.99 (0.94)	1.92 (0.88)	1.92 (0.87)	2.01 (0.93)	1.98 (0.96)	1.93 (0.96)
Self-esteem prob.^a^ (n = 2275)	1.95 (0.52)	1.93 (0.53)	1.96 (0.52)	1.96 (0.52)	1.95 (0.54)	1.93 (0.55)	1.88 (0.50)
Attention prob.^a^ (n = 2289)	1.90 (0.55)	2.06 (0.59)	1.87 (0.54)	1.86 (0.54)	2.03 (0.60)	1.93 (0.61)	2.14 (0.58)
Reading and writing prob.^a^ (n = 2210)	1.58 (0.88)	1.78 (0.94)	1.55 (0.86)	1.55 (0.86)	1.74 (0.92)	1.57 (0.78)	1.88 (0.99)
Bullied^a^ (n = 2278)	1.17 (0.42)	1.36 (0.59)	1.12 (0.37)	1.12 (0.37)	1.34 (0.55)	1.12 (0.36)	1.41 (0.67)
Parent`s marital sit.^b^ (n = 2257)							
Together	1823 (80.8)	309 (78.0)	1514 (81.3)	1441 (81.6)	228 (79.7)	73 (76.8)	81 (73.6)
Separated or divorced	434 (19.2)	87 (22.0)	347 (18.7)	325 (18.4)	58 (20.3)	22 (23.2)	29 (26.4)
Alcohol intoxication^b^ (n = 2246)							
Never	1646 (73.3)	260 (66.8)	1386 (74.6)	1327 (75.2)	200 (71.4)	59 (64.2)	60 (55.0)
1–10	501 (22.3)	93 (23.9)	408 (22.0)	380 (21.5)	60 (21.4)	28 (30.4)	33 (30.3)
> 10	99 (4.4)	36 (9.3)	63 (3.4)	58 (3.3)	20 (7.2)	5 (5.4)	16 (14.7)
Smoking^b^ (n = 2248)							
No	2001 (89.0)	325 (83.6)	1676 (90.2)	1598 (90.6)	236 (84.3)	78 (81.7)	89 (81.7)
Yes	247 (11.0)	64 (16.5)	183 (9.8)	166 (9.4)	44 (15.7)	17 (18.4)	20 (18.3)

For each variable, the number of participants with available data is shown in parenthesis

*YH1* Young-HUNT1, *PA* physical aggression

^a^Continuous variables

^b^Dichotomous variables

### Cross-Sectional Correlates of Physical Aggression in Early Adolescence

Results of the logistic regression analysis with physical aggression in YH1 as the dependent variable and the risk factors assessed at YH1 as correlates are presented in Table 3. The ORs show that younger age, male sex, anxiety and depression, loneliness, low self-esteem, attention problems, reading and writing problems, being bullied, drinking alcohol, and smoking were all associated with physical aggression in early adolescence (Model 1). The largest ORs were for male sex (OR 10.04, 95% CI [7.45, 13.52]) and being drunk more than 10 times (OR 8.08, 95% CI [4.60, 14.17]). Parent’s marital status was not associated with physical aggression in YH1. When adjusting for all the correlates in the fully adjusted model, anxiety and depression, loneliness, and self-esteem problems were not significantly associated with physical aggression in YH1.

**Table 3:** Logistic regression to identify correlates of physical aggression in YH1

	Model 1		Model 2	
	ORs	95% CI	ORs	95% CI
Age	0.85*	[0.75, 0.97]	0.62***	[0.52, 0.73]
Sex				
Boys (ref = Girls)	10.04***	[7.45, 13.52]	12.74***	[8.98, 18.08]
Anxiety and depression	2.04***	[1.55, 2.69]	1.08	[0.74, 1.58]
Loneliness	1.28***	[1.12, 1.46]	0.99	[0.84, 1.18]
Self-esteem prob	1.72***	[1.35, 2.19]	1.15	[0.85, 1.54]
Attention prob	2.39***	[1.93, 2.95]	1.76***	[1.38, 2.26]
Reading and writing prob	1.29***	[1.14, 1.47]	1.16*	[1.01, 1.33]
Bullied	2.44***	[1.92, 3.09]	2.17***	[1.65, 2.85]
Parent’s marital sit				
Separated or divorced (ref = Together)	1.32	[0.99, 1.76]	0.89	[0.64, 1.23]
Alcohol intoxication				
1–10 (ref = Never)	2.01***	[1.47, 2.75]	1.54*	[1.08, 2.21]
> 10 (ref = Never)	8.08***	[4.60, 14.17]	4.55***	[2.38, 8.69]
Smoking				
Yes (ref = No)	3.39***	[2.33, 4.93]	1.78*	[1.12, 2.81]

Model 1—Associations for correlates and physical aggression adjusted for age in YH1 and sex. Model 2—Fully adjusted model. All risk factors and covariates (age, sex) are included in the model. n = 2289

*ORs* Odds Ratios, *CI* Confidence Interval, *YH1* Young-HUNT1

*p < 0.05, **p < 0.01, ***p < 0.001

### Risk Factors for Physical Aggression Starting in Late Adolescence

Results from the multinomial logistic regression analysis with *the late adolescent aggression group* as the dependent variable and *the non-aggression group* as the reference group are presented in Table 4. When adjusting for age and sex (Model 1), the results showed that male gender (OR 4.46, 95% CI [2.85, 6.99]), attention problems (OR 1.56, 95% CI [1.08, 2.67]), alcohol use (alcohol intoxications between 1 to 10 times, OR 2.46, 95% CI [1.50, 4.15], and more than 10 times, OR 4.13, 95% CI [1.44, 11.83]), and smoking (OR 3.49, 95% CI [1.92, 6.35]) in early adolescence were statistical significant risk factors for onset of physical fighting in late adolescence. In the fully adjusted model (Model 2), male gender, alcohol intoxications between 1 and 10 times, and smoking remained statistic significant.

**Table 4:** Multinomial logistic regression to identify risk factors in YH1 for different aggression groups

	The late adolescent aggression group vs the non-aggression group				The persistent aggression group vs the desisting aggression group			
	Model 1		Model 2		Model 1		Model 2	
	ORs	95% CI	ORs	95% CI	ORs	95% CI	ORs	95% CI
Age	0.90	[0.71, 1.13]	0.76	[0.57, 1.01]	1.06	[0.83, 1.36]	0.75	[0.55, 1.01]
Sex								
Boys (ref = Girls)	4.46***	[2.85, 6.99]	6.32***	[3.72, 10.72]	2.22*	[1.05, 4.71]	1.98	[0.88, 4.42]
Anxiety and depression	1.57	[0.97, 2.56]	1.07	[0.56, 2.07]	1.03	[0.64, 1.67]	0.98	[0.50, 1.94]
Loneliness	1.23	[0.97, 1.54]	1.13	[0.84, 1.51]	0.93	[0.73, 1.19]	0.88	[0.65, 1.19]
Self-esteem prob	1.44	[0.95, 2.20]	1.08	[0.65, 1.80]	0.86	[0.55, 1.36]	0.65	[0.38, 1.11]
Attention prob	1.56*	[1.08, 2.67]	1.14	[0.73, 1.77]	1.35	[0.94, 1.92]	1.34	[0.88, 2.02]
Reading and writing prob	1.03	[0.80, 1.32]	0.92	[0.70, 1.21]	1.14	[0.92, 1.42]	1.09	[0.86, 1.38]
Bullied	0.87	[0.48, 1.56]	0.92	[0.50, 1.71]	1.14	[0.81, 1.61]	1.15	[0.77, 1.72]
Parent`s marital sit								
Separated or divorced (ref = Together)	1.43	[0.87, 2.35]	1.36	[0.81, 2.29]	1.43	[0.86, 2.39]	1.35	[0.77, 2.37]
Alcohol intoxication								
1–10 times (ref = Never)	2.46**	[1.50, 4.15]	1.86*	[1.04, 3.34]	2.35**	[1.33, 4.14]	2.78**	[1.49, 5.17]
> 10 times (ref = Never)	4.13**	[1.44, 11.83]	1.91	[0.59, 6.19]	4.32**	[1.85, 10.10]	5.35**	[1.99, 14.37]
Smoking								
Yes (ref = No)	3.49***	[1.92, 6.35]	2.43*	[1.21, 4.90]	1.31	[0.71, 2.43]	0.69	[0.33, 1.46]

Model 1—Associations for risk factors and physical aggression adjusted for age in YH1 and sex. Model 2—Fully adjusted model. All risk factors and covariates (age, sex) are included in the model. *The non-aggression group* n = 1793*, the desisting aggression group* n = 287*, the late adolescent aggression group* n = 98*, and the persistent aggression group* n = 111

*ORs* Odds Ratios, *CI* Confidence Interval, *YH1* Young-HUNT1

*p < 0.05, **p < 0.01, ***p < 0.001

### Risk Factors for Persistent Physical Aggression During Adolescence

Results from the multinomial logistic regression analysis with *the persistent aggression group* as the dependent variable and *the desisting aggression group* as the reference group are presented in Table 4. When only adjusting for age and sex (Model 1), the results showed that male gender (OR 2.22, 95% CI [1.05, 4.71]) and alcohol intoxications (between 1 and 10 times, OR 2.35, 95% CI [1.33, 4.14] and more than 10 times, OR 4.32, 95% CI [1.85, 10.10]) increased the risk of persistent aggressive behavior, relative to desisting aggressive behavior. Male gender and getting drunk between 1 and 10 times both doubled the odds of continued physical aggressive behavior during adolescence, compared to desisting aggressive behavior, whereas getting drunk more than 10 times increased the odds by four. The OR for male gender attenuated in the fully adjusted model (Model 2), while the ORs for alcohol intoxication between 1 and 10 times and more than 10 times remained statistic significant, indicating a strong predictive effect of alcohol drinking in early adolescence for persistent physical aggression during adolescence.

## Discussion

In this study, we used the data from the Young-HUNT study to examine correlates of physical aggression in early adolescence and examine risk factors for physical aggression during the transition from early to late adolescence. Our results showed that younger age, male sex, anxiety and depression symptoms, loneliness, self-esteem problems, attention problems, reading and writing problems, being bullied, alcohol intoxications, and smoking were all associated with physical aggression in early adolescence. Male sex, having attention problems, and drinking alcohol and smoking cigarettes in early adolescence (mean age 14.5 years) increased the risk of emerging physical aggression in late adolescence (mean age 18.4 years), and only male sex and frequent alcohol intoxications were related to increased risk of continuing physical aggression from early to late adolescence.

Our results concerning correlates of physical aggression in early adolescence were consistent with the literature [17–20, 28, 39]. While some of the associations attenuated in the fully adjusted model, attention problems, reading and writing problems, being bullied, alcohol intoxication frequency between 1 and 10 times and more than 10 times, and smoking still were significantly associated. The strongest associations were between being drunk more than 10 times, being bullied, and having attention problems and physical aggression. Having divorced parents was the only variable not associated with physical aggression in early adolescence. One possible reason for the attenuating effect of anxiety and depression could be the combining of anxiety and depression problems in the same scale. Previous findings have shown considerable evidence for the association between depression and physical aggression [20], while the association with anxiety is more ambiguous, such that anxiety is shown to be both associated with [21] and protective against aggression [25]. Both high and low self-esteem have been found to correlate with physical aggression [29], which could be an explanation for the attenuated effect of low self-esteem in the fully adjusted, more conservative model. Having friends and participating in social activities are for many the most important part of adolescence. Being bullied has a strong association with physical aggression in early adolescence in our study. It could be that the effect of loneliness was attenuated in the fully adjusted model because bullying was a better predictor and accounted for the variance captured by loneliness. Also, experiencing loneliness could be linked to other factors, for example, lack of mutual interests, that are not necessarily associated with bullying or peer rejection and thus do not increase the risk for physical aggression.

The prevalence of physical aggression among adolescents in the present study was 17.4% in early adolescence and 9.1% in late adolescence, which is somewhat lower than the prevalence in an American population sample [1] but still represents a profound clinical and societal challenge. The proportion in the high persistent aggression group (4.9%) resembles high aggression groups in previous studies [3, 10, 15]. However, given differences in sample characteristics and aggression measurements between previous studies and ours, a direct comparison of our aggression courses and those in previous studies is not possible.

Our main goal in this study was to examine if risk factors in early adolescence could help explain why non-aggressive adolescents started displaying physical aggression in late adolescence, and why some adolescents continued with physically aggressive behavior while others desisted. 4.3% of the sample reported onset of physical aggression in late adolescence, which is consistent with previous studies identifying a small group of adolescents with aggressive behavior starting in adolescence [15, 31]. Our results showed that boys with attention problems and substance use (e.g. heavy drinking and smoking) in early adolescence had an increased risk of physical fighting, 4 years later in late adolescence. The predictive effect of having attention problems attenuated in the fully adjusted model, but the effect of male sex, alcohol drinking (intoxications 1–10 times), and smoking remained strong. The predictive effect of delinquent behaviors, such as drinking and smoking, early in adolescence on physical aggression starting in late adolescence could be understood in the context of the specific characteristics of the adolescent period [15, 48, 49]. During the transition to adulthood, the influence of friends typically increases, while parental supervision decreases. Drinking alcohol and smoking in early adolescence could be associated with involvement with deviant friends, providing an accepting environment for norm-breaking behavior, which over time can increase the risk for behaviors such as physical fighting. ADHD symptoms have been shown to have a predictive effect on physical aggression [3, 5, 22]. Our results indicate that attention problems are of some relevance, but they are not an important predictor in distinguishing between non-aggressive behavior and physical aggression starting in late adolescence. It could be that the emergence of physical aggression in late adolescence is accounted for by alcohol use and smoking.

Knowing the negative consequences for the victims and the perpetrators, it is important to be able to identify the group with highly persistent aggressive behavior and know what separates this group from the other courses of aggression. In terms of developing interventions aimed at stopping physically aggressive behavior in adolescence, it is especially interesting to know what separates persistence from desistance of physical aggression. Our results showed that male sex and frequency of alcohol intoxication increased the risk of persistent aggressive behavior during adolescence but that the effect of male sex was attenuated when all of the risk factors were included, leaving heavy drinking as the sole important predictive factor separating adolescents who followed a course of persistent aggression from those who followed a course of desisting aggression. Results indicated a dose–response relationship between alcohol and aggression, as shown in previous research [43]. Recent studies on alcohol consumption have shown a declining trend in alcohol drinking among adolescents [61], which could result in declining physical aggression. However, it is unclear if the declining trend applies to all levels of consumption, especially the heavy drinking.

Results from this study regarding sex and overall age trends in physically aggressive behavior are consistent with previous studies [6, 9–11, 13, 15, 16]. Our results show that the prevalence of physical aggression is higher in boys than in girls and that more boys than girls are in the persistent aggression group. The prevalence of physical aggression decreased from early adolescence to late adolescence, and lower age was associated with an increased risk of physical aggression in the cross-sectional analysis. These findings are in line with previous studies showing a decrease in physical aggression with increasing age, thereby supporting the understanding that physical aggression is a normative part of children’s development and that as children get older and mature their physical aggressive behavior decreases [11].

## Clinical Implications

Physical aggression during childhood and adolescence is associated with many different risk factors, and the risk factors vary for different developmental periods. For interventions in adolescence to be effective, they need to target the relevant risk factors. Our results showed that heavy alcohol drinking in early adolescence is concurrently and longitudinally associated with aggressive behavior. Excessive alcohol drinking predicted the onset of aggressive behavior starting in late adolescence and separated persistent from desisting physical aggressive behavior over the later adolescent years. These findings highlight the need for early screening and assessment of alcohol drinking in adolescence. Taken together, the results indicate the need to identify adolescents at risk, with a specific focus on boys, as early as possible. Prevention efforts, in general, could focus on reducing alcohol drinking and smoking in early adolescence, while prevention efforts in schools could focus on facilitating mastery for students with attention and learning difficulties and on preventing bullying.

## Strengths and Limitations

The major strength of the current study is the longitudinal design, the large population-based sample with a high response rate, and a dataset that allows for investigation of a wide array of correlates. In this study, we chose to examine the development of physical aggression from early to late adolescence. Examining the same adolescents at two timepoints (YH1 and YH2), four years apart, provides important information on the different courses of physical aggression in adolescence and corresponding risk factors.

A limitation of the study is that the measurement for physical aggression is based on only one item assessing engagement in physical fights. However, one- item measurements of physical aggression have been used in other studies [62–64], and while more nuanced measures of aggression are commonly used in clinical populations with smaller samples, they are less used in large population-based surveys. Other forms of physical aggression were not captured in this study, which may have resulted in the lower prevalence of physical aggression in this study and affected the associations we observed. Another limitation is shared method variance, as both the dependent variable physical aggression and the risk factors were measured by self-report questionnaire and completed by the same informant. Also, we had only two timepoints (YH1 and YH2) to base the aggression courses on. Additional timepoints would allow us to chart different aggression trajectories using more sophisticated, person-centered approaches (e.g., latent class growth analysis) to better probe the individual differences in aggression trajectories. Previous research suggests that SES is associated with physical aggression [10]. Our analyses adjusted for sex and age but not SES, which is a limitation. The questionnaire includes a broad range of physical and mental health variables and lifestyle factors. None of the scales or items have diagnostic precision but assess symptoms and problems. Some of the scales are shortened versions of original screening instruments, although shown to be reliable in previous studies [57, 58].

## Summary

We identified several strong correlates of physical aggression in early adolescence including male gender, attention problems, reading and writing problems, being bullied, intensive alcohol drinking, and smoking. Different risk factors were predictive of different courses of physical aggression. For example, being male and engaging in heavy drinking and smoking during young adolescence were important predictors of physically aggressive behavior starting in late adolescence, while intensive alcohol drinking was predictive of persistent versus desisting use of physical aggression from early to late adolescence. The findings provide insights that can inform the development of preventative measures and therapeutic interventions targeting physical aggression in adolescence.
